# Stimulus material selection for the Dutch famous faces test for older adults

**DOI:** 10.3389/fmed.2023.1124986

**Published:** 2023-04-14

**Authors:** Evi H. T. van den Elzen, Yvonne Brehmer, Katrijn Van Deun, Ruth E. Mark

**Affiliations:** ^1^Department of Developmental Psychology, Tilburg University, Tilburg, Netherlands; ^2^Department of Cognitive Neuropsychology, Tilburg University, Tilburg, Netherlands; ^3^Aging Research Center, Karolinska Institutet, Stockholm, Sweden; ^4^Department of Methodology and Statistics, Tilburg University, Tilburg, Netherlands

**Keywords:** aging, famous faces, famous names, memory, naming, preclinical AD, recollection

## Abstract

Worldwide, approximately 22% of all individuals aged 50 years and older are currently estimated to fall somewhere on the Alzheimer’s disease (AD) continuum, which can be roughly divided into preclinical AD, mild cognitive impairment (MCI), and AD dementia. While episodic memory loss (among other aspects) is typically required for a diagnosis of AD dementia, MCI is said to have occurred when cognitive impairment (including memory loss) is worse than expected for the person’s age but not enough to be classified as dementia. On the other hand, preclinical AD can currently only be detected using biomarkers; clinical symptoms are not apparent using traditional neuropsychological tests. The main aim of the current paper was to explore the possibility of a test which could distinguish preclinical AD from normal aging. Recent scientific evidence suggests that the Famous Faces Test (FFT) could differentiate preclinical AD from normal aging up to 5 years before a clinical AD diagnosis. Problematic with existing FFTs is the selection of stimulus material. Faces famous in a specific country and a specific decade might not be equally famous for individuals in another country or indeed for people of different ages. The current article describes how famous faces were systematically selected and chosen for the Dutch older (60+) population using five steps. The goal was to design and develop short versions of the FFT for Dutch older adults of equivalent mean difficulty. In future work, these nine parallel versions will be necessary for (a) cross-sectional comparison as well as subsequent longitudinal assessment of cognitively normal and clinical groups and (b) creating personalized norms for the normal aged controls that could be used to compare performance within individuals with clinical diagnoses. The field needs a simple, cognitive test which can distinguish the earliest stages of the dementia continuum from normal aging.

## Introduction

Worldwide, 416 million older adults are currently estimated to fall somewhere on the Alzheimer’s disease (AD) continuum, corresponding to approximately 22% of all individuals aged 50 years and older (1). This continuum can be roughly divided into three stages, namely: preclinical AD, mild cognitive impairment (MCI), and clinical AD dementia. Preclinical AD typically begins in midlife (2, 3) and is characterized by pathological processes in the brain including, for example, abnormal levels of peptide amyloid-beta Aβ (Aβ+; 3, 4). At this stage cognitive impairment is usually not apparent and cognitive tests currently employed in clinical practice fail to detect it (5). As there are large between-person differences in cognitive abilities as well as within-person change over time, distinguishing cognitive impairment due to preclinical AD from that due to normal aging is difficult (6).

Currently, preclinical AD can only be detected via the assessments of biomarkers (3, 4), which are invasive, can be painful (e.g., cerebrospinal fluid needs to be tapped using a lumbar puncture), tend to be costly (e.g., PET/MRI scans) and are not widely available. In addition, noticeable biomarkers are also present in approximately 50% of healthy, cognitively normal (CN) older adults, many of whom never progress to clinical AD (7). Furthermore, many barriers exist toward (timely) AD diagnosis at the patient, caregiver, health care, and societal level, with many individuals being diagnosed too late or not at all (8–10). Research has also suggested that most (85–95%) CN older adults would still want to receive the AD diagnosis, either for themselves or for their partner, despite the fact that no cure for AD is available as yet (11, 12). One solution to these difficulties currently facing timely AD diagnosis could be an inexpensive, widely available cognitive screening test that could accurately distinguish preclinical AD from healthy aging while also taking individual differences into account.

Clinical AD dementia is typically determined according to established guidelines, for example, using the Diagnostic and Statistical Manual of Mental Disorders, Fifth Edition (DSM-V; 13) with episodic memory loss a key symptom. Traditional episodic memory tests are not sensitive enough to detect preclinical AD (14, 15). “Normal” performance on standard episodic memory tests could lead to the erroneous conclusion that cognition is normal at the preclinical AD stage.

While some controversy exists, semantic memory performance is generally believed to remain preserved, at least until the later stages in the AD continuum (13, 16). However, in some studies, a steep decline in semantic memory performance was observed approximately 5 years prior to clinical AD diagnosis (15, 17). Furthermore, persons with preclinical AD often experience difficulty with verbal abilities, including category fluency for living things (e.g., animals), and the naming of famous people (18, 19). These findings suggest that a more detailed exploration of between-person differences in semantic memory performance could be fruitful when attempting to design a cognitive screening instrument sensitive enough to detect preclinical AD. This test, if it can distinguish normal aging from preclinical AD and/or MCI (between-person differences), could also be used to explore within-person change over time.

The Famous Face Test (FFT) could be the ideal screening tool to detect within-person change during the earliest stages of AD, while also considering between-person differences in level and change. In a standard FFT, participants are shown photographs of famous faces and then asked to name each face (20, 21). Different versions of the FFT exist that encompass a range of different photographs, all representing famous persons from different decades and categories (e.g., actors, athletes, musicians, media personalities, and/or politicians). Problematic with all existing FFTs is the selection of stimulus material. To our knowledge, this has never been systematically done for the population of interest (e.g., older adults), or indeed the country of origin (including, for example nationally and internationally famous faces). Most FFTs are not standardized in any way. Many of these tests use, for example, photographs with props/backgrounds which could make identification of the famous faces easier, and many do not report where their stimuli were sourced.

Previous research has investigated whether or not healthy younger and older adults recognized famous faces (e.g., 22–24). However, only four studies have explored FFTs in clinical samples of individuals diagnosed with either mild MCI or preclinical AD based on biomarker evidence or a clinical AD diagnosis at two-year follow up (25–28). In general, these studies found that these individuals performed more poorly on an FFT than CN older adults, despite seemingly normal performance on other tests of global cognition, memory, and fluency. This led these authors to conclude that the FFT can detect the earliest stages of AD. More specifically, individuals with mild MCI or preclinical AD perform more poorly on the naming of recent or current famous individuals, whereas naming of remote famous faces (individuals who were famous a long time ago) did not differ from CN older adults (26, 29). This implies a temporal gradient that may characterize the earliest signs of cognitive decline in AD, and that can be measured using the FFT.

There are many limitations however to these previous studies including: different items used across studies making comparison difficult, insufficient quality of photographs, and stimuli not standardized or selected for the population studied. Mostly, tasks included less than 50 famous faces that were not developed in a systematic manner (or not obviously so from the methodology described in the respective publications). Stimulus material often was not standardized, including props that indicate who the person was (e.g., a U.S. flag on the background of a U.S. president’s photograph) In addition, while all were conducted in different countries and in specific populations (e.g., university students or older adults) previous FFT versions were not country-specific, nor tailored to the population of interest. Ideally, the FFT should also be suitable for longitudinal assessment in both cognitively normal and clinical groups, for example by means of different short FFT versions of equivalent mean difficulty. In the current study, the selection of stimulus material and development of nine short versions of the first Dutch FFT (D-FFT) is described that will be used at a later stage in this ongoing project for longitudinal assessment in both cognitively normal and clinical populations. By not only ensuring equivalent mean difficulty levels, but also that each version has an equal number of stimuli from each decade, the described temporal gradient can also be assessed in future steps as well.

Previous studies using an FFT furthermore included different tasks such as recall (recollection of the name of the famous person), identification [providing person-identity semantic knowledge (PISK) about the famous person], and recognition [indicating whether the person was famous, or choosing their name out of multiple options] (22–29). In a recall task, individuals can only rely on recollection, which involves the active recovery of information and is also referred to as remembering (30), which is more sensitive to the earliest stages of AD than familiarity, as the latter involves a feeling of knowing without remembering (30, 31). As floor effects are common on an FFT recall task in clinical populations, the D-FFT also includes a multiple-choice recognition task, where participants can rely on familiarity in addition to or instead of recollection. This recognition task, while not used to determine stimulus selection in this paper, may be especially informative in clinical populations in future steps of this project.

**Figure 1:**
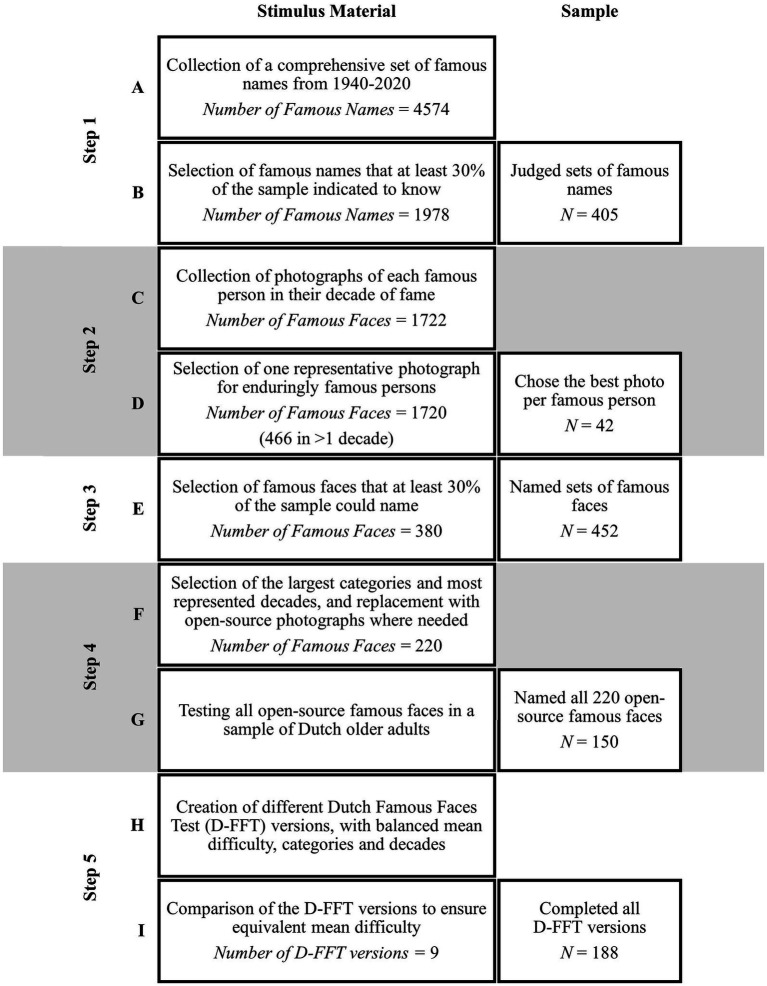
Flowchart of steps 1–5. Each step had one or more sub-steps, which are described together with the resulting number of famous names/faces. Each step included at least one sub-step that involved data collection, of which sample size and the main task are described. Further sample descriptive statistics are presented in Table 2.

The aim of the current study was to develop different versions of the first Dutch FFT specifically for older adults aged 60 years and above, to be able in the future to conduct a (a) cross-sectional comparison as well as subsequent longitudinal assessment of cognitively normal and clinical groups and also be able to (b) create personalized norms for the normal aged controls that could be used to compare performance within individuals with clinical diagnoses. The development of this new test and its nine versions was conducted in five steps. These were as follows: (1) selecting famous names, (2) constructing a database containing one representative photograph per famous person, (3) selecting famous faces that Dutch older adults could indeed name, (4) developing different versions of the D-FFT, and (5) testing these D-FFT versions for equivalent mean difficulty. An overview of the five steps used to achieve these aims is presented in Figure 1.

## General methods

### Participants

Every step of the selection process included some form of data collection with different (partly overlapping) samples of Dutch older adults. All Dutch-speaking older adults aged 60 and older could participate in this study if they had access to the internet on a computer, tablet, or smartphone. See Table 1 for the descriptive statistics for the samples assessed in each of the five steps.

**Table 1:** Descriptive statistics of participants in steps 1–5.

Step	*N*	Age			Female (%)	Education level (%)			Overlap (%)^4^
		*M*	*SD*	Range		Low^1^	Middle^2^	High^3^	
1	405	69.84	5.98	60–91	62	5	17	78	
2	42	68.67	4.82	60–81	69	0	26	74	88
3	452	70.90	5.64	60–92	61	7	20	73	60
4	150	70.56	6.17	60–90	60	9	19	72	66
5	188	70.31	5.83	60–85	60	9	23	68	58

^1^Low education: Finished low-level secondary education or less. ^2^Middle education: Finished average-level secondary education. ^3^High education: Finished high-level secondary education or has a University degree. ^4^The proportion of participants who had also participated in (a) previous step (s) is reported (Overlap %). Participants in step 5 could not have participated in step 4 as well.

Participants were recruited via the researchers’ personal networks, social media, and different types of elderly associations that distributed the study invitation in their (online) newsletters. Such elderly associations included Higher Education for Elderly (Hoger Onderwijs voor Ouderen, HOVO) and local elderly organizations spread all over the Netherlands (e.g., Catholic Associations of Elderly/Katholieke Bond van Ouderen, KBO). This study was approved by the ethical review board of the Tilburg School of Social and Behavioral Sciences at Tilburg University (RP609) on 7 September 2021. All participants were informed about the study and gave written informed consent before participation.

### Procedure

For all five steps, individuals indicated their interest to participate by filling in their email address, date of birth and (if they required more information) phone number using an online form. After registering, participants received an email with an information letter and personal link to the online task. They could complete all steps of this study on a PC (desktop or laptop), tablet, or smartphone, but were encouraged to use a PC due to the larger screen and keyboard.

Participants’ registrations and subsequent data for all steps of this study were collected using Qualtrics Experience Management (EM) software (32). Participants answered all questions by pressing the button for the answer of their choice or, if applicable, typing their answers in a box and then pressing a button to continue. Although each step contained specific questions (e.g., questions about person-identity semantic knowledge, a recognition task, or confidence ratings), only the questions that were relevant for the selection of stimulus material will be reported here.

In each step, participants were presented with one famous name (steps 1 and 2) or famous face (steps 3 to 5) at a time. Famous names and faces were presented on a white background, in the middle of the screen of the participant’s device of choice. Participants were always asked the first question of the task on the same page, right below the famous name or famous face. In case there were follow-up questions, the famous name or famous face would remain visible at the top of the screen. All calculations and analyses were performed using R version 4.2.1 (33) and RStudio version 2022.07.01 (34).

In steps 3 to 5, participants completed a multiple-choice recognition task directly after the recall task, in which they would see all famous faces again but now chose the correct name out of multiple options, respectively. However, recognition performance over the 1,720 items in step 3 was near-ceiling (*M*_recognition_ = 83.37%, *SD* = 15.10%). Therefore, only the recall task was used for stimulus selection and the multiple-choice recognition task is not described in further detail in the current paper.

### Statistical analysis

In steps 4 and 5, different versions were created and tested for equivalence of mean difficulty level. The sample of 338 persons was randomly split in two groups, stratified in terms of age, gender, and education, resulting in a sample of 150 for creating the D-FFT versions and 188 for testing equivalence of the mean difficulty level. First, to create the D-FFT versions, we used the following procedure in step 4: One hundred thousand sets of 10 versions were generated by randomly distributing the items over the (non-overlapping) D-FFT versions under the constraints set to obtain an equal number of items per decade and category in each version; the R software language for statistical computing was used for generating the versions. For each of the generated sets, the mean proportion of correct naming responses was obtained for each of the D-FFT versions in the set, using the data of the 150 participants. The set with the smallest difference between the largest and smallest mean proportion of correct naming responses was then retained to create the parallel versions. Second, the versions of this set were tested for equivalence on the remaining set of 188 participants in step 5. Each of the participants completed all items and versions, hence a repeated measures analysis applies in which we treated the version as a fixed factor and the participant as a random factor. The lme4 R package was used for the general linear mixed model analysis (35).

## Step 1: Selecting famous names

Step 1’s aim was (a) to collect a comprehensive set of famous peoples’ names from 1940 to 2020 (decades of relevance for older adults) and (b) to test Dutch older adults on these famous names and then select the names which they recognized for the next steps of this study.

### A. Collection of a comprehensive set of famous names from 1940 to 2020

The systematic collection of famous names was based on the Pantheon 1.0 dataset of globally famous biographies that are categorized by occupation (36). Based on the Pantheon 1.0, 10 overarching categories were constructed, namely: Artists, Business & Law, Explorers, Film & Theater, Institutions, Literature & Science, Politicians, Public Figures, Singers & Musicians, and Sports. An overview of all categories and the number of Dutch and international famous names is presented in Table 2. After setting up the categories, famous names were searched for in two steps. First, the Dutch Wikipedia’s yearly news overviews were searched for every year between 1940 and 2020. All famous names that appeared in these general Dutch news overviews were assigned to the correct category and decade(s) in which they occurred (1940–2020s). If famous names occurred in different categories or in multiple decades, they were assigned to several categories and decades. Second, the names in each category and decade were complemented by collecting the famous names from category-specific recurring events, both globally and specifically for the Netherlands. Extracting those names from Dutch Wikipedia pages led to a systematic collection of one or multiple names for each occurrence of the event. Examples include: winners of the FIFA world and Dutch premier league soccer player(s) of the year. A complete overview of all sources per category and the corresponding years can be found in the Supplementary material. This search led to a total of 4,574 unique famous names. The number of names per category, national and international, is presented in Table 2.

**Table 2:** Overview of categories and the corresponding Dutch and international number of names in step 1.

	Number of names		
Category	Dutch	International	Total
Art	80	36	116
Business and law	23	45	68
Film and theater	256	408	664
Institutions (e.g., Royals)	43	147	190
Literature and science	180	127	307
Public figures	149	166	315
Politics	245	541	786
Singers and musicians	399	680	1,079
Sports	483	602	1,085

The total number of famous names adds up to over 4,574 famous names (i.e., 4,610), as several famous names were assigned to multiple categories.

### B. Selection of famous names that Dutch older adults indicated to know

In total, 405 older adults (see Table 1 for sample descriptive statistics) rated the 4,574 unique famous names that were collected indicating if they know this person (“Yes” or “No”). If they did not know the name (i.e., pressed “No”) or did not answer within 10 s, they automatically proceeded to the next famous name.

As the total set of 4,574 famous names was too long for one participant to work through, each participant saw one set of 250 randomly selected names which they could complete in one 45-min session. Selection of names that were presented to each participant was achieved using Qualtrics software (32). Participants could decide to do more sets of 250 words each (up to a maximum of 4) in one session. Data collection ended when each of the 4,574 famous faces had been rated by at least 20 participants.

**Figure 2:**
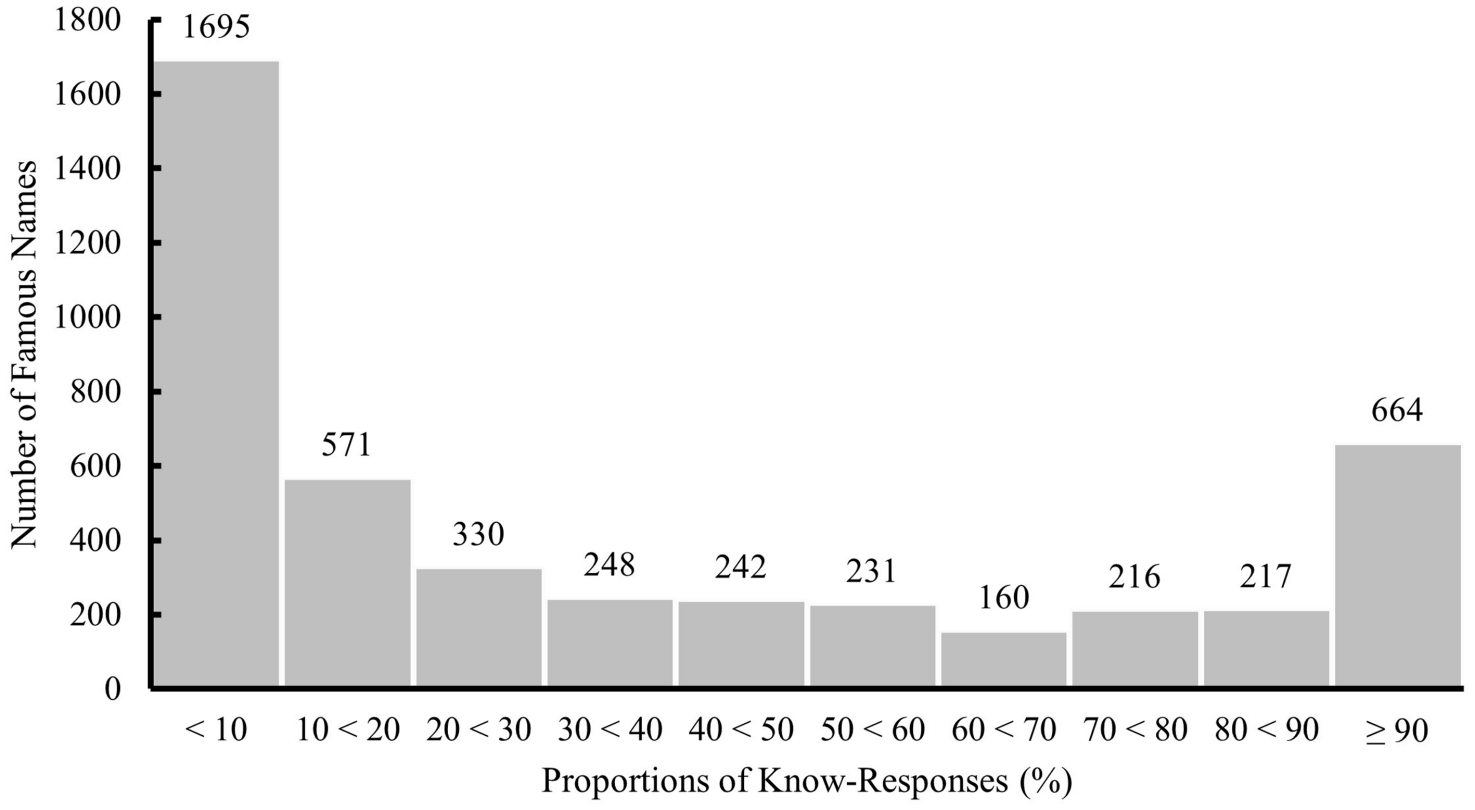
Distribution of proportions of know-responses to all 4,574 famous names evaluated in step 1. Labels on top of each bar indicate the number of famous names per interval.

For each of the 4,574 famous names, the number of times a participant indicated to know the name was calculated, relative to the total number of responses provided for this famous name. To illustrate, if exactly 20 participants responded to a famous name and 10 of them indicated to know the name, the calculated percentage was 50%. For the distribution of know-responses per famous name, see Figure 2. In this and following steps, we used a 30% cut-off to ensure enough variation in difficulty of stimulus material in the D-FFT. This means that 30% of participants who responded to the name should have stated that they knew it in order for that name to make it into the next step. As a result, the initial pool of 4,574 famous names was reduced to 1,978.

## Step 2: Selecting photographs of the famous names 30% of participants stated that they knew in step 1

For the 1,978 famous names which remained after step 1 we focused on constructing a database of representative photographs, one for each famous person. Importantly, the photographs needed to be taken in the decade when the person became/was famous, not a contemporary one. In addition, specifically Dutch older adults needed to find the photograph representative and recognizable. The aim of step 2 of this study was (c) to collect representative photographs of the 1,978 selected famous names from step 1, and (d) to select the most representative photograph of famous persons who were famous in more than one decade.

### C. Collection of photograph(s) of each famous person in their decade of fame

For the 1,978 famous names which resulted from step 1 we focused on constructing a database of representative photographs, one for each famous person. Portrait photographs were collected for each of the 1,978 famous names in (each of) the decade(s) when they were famous. For famous persons who remained famous for more than one decade (hereafter labeled “enduring”), one photograph was collected for each decade they were famous in. For famous persons who were famous during only one decade (hereafter labeled “transient”), one photograph was collected that was taken in the specific decade they were famous in.

**Figure 3:**
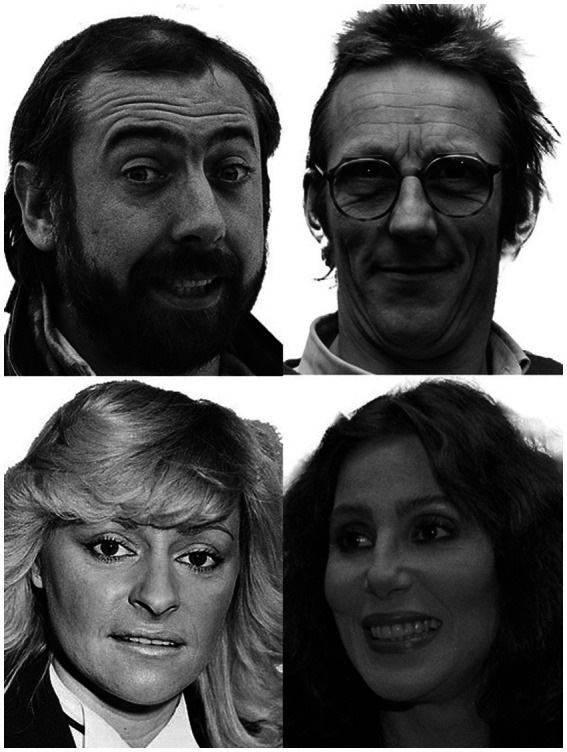
Examples of test material of the D-FFT. The famous faces depicted here are part of the practice trial in steps 4 and 5 and are not included in the nine D-FFT versions. These photographs have a CC0 license (https://creativecommons.org/share-your-work/public-domain/cc0/) or are in the Public Domain and do therefore not require a consent statement.

Photographs could be found anywhere on the internet, but they needed to meet the inclusion criteria listed in Table 3. Ideally, the photograph also had an open-source creative commons (CC) license, so that it could be adapted and shared in a later stage. As photographs were standardized and thereby adapted, all CC licenses were accepted except for NoDerivatives (ND), as these may not be shared in adapted form. Hence, photographs with one of the following CC licenses could be included: Public domain or No Rights Reserved (CC0), Attribution (CC BY), Attribution Share Alike (CC BY-SA), Attribution-Non-Commercial (CC BY-NC), and Attribution-Non-Commercial-ShareAlike (CC BY-NC-SA). If multiple photographs were found of the same famous person in the same decade, the photograph that best matched the inclusion criteria was chosen. Of the 1,978 famous persons, 256 were excluded, because no photograph was found that met the inclusion criteria. Of the 1,722 remaining famous persons, 468 were enduringly famous and had multiple photographs from different decades. The other 1,254 transiently famous persons had one photograph. Using Photoshop, all famous faces were cut out and placed on a white background; the photograph was converted to black and white; and all photographs were resized to 250 by 333 pixels. In this way, all photographs were now in a standardized format. See Figure 3 for some example items.

**Table 3:** Inclusion criteria for photographs of famous faces.

Inclusion criteria
1. Sufficient size and quality, such that the face could be cut out and the remaining photograph would be of sufficient quality (i.e., at least 250 × 333 pixels).
2. Taken from the front (e.g., not side profile).
3. As few accessories as possible, but at least: no visible props (e.g., American flag, microphone, etc.) or accessories that could provide clues as to who the person was.
4. The photograph had to be dated and the photograph had to be taken within the decade that the person was famous.

### D. Selection of the one representative photograph for enduringly famous persons

In total, 42 older adults participated in step 2 (see Table 1 for sample descriptive statistics). Stimulus material consisted of both the famous names that were selected in step 1 and the collected photographs of each famous face. For each famous name, participants saw the question: Do you know this person? (“Yes” or “No”). If they did not know the name (i.e., pressed “No”) or did not answer within 10 s, they automatically proceeded to the next famous name. If they indicated to know the name, they saw the next question: Can you visualize this person’s face? (“Yes” or “No”). If they indicated they could visualize the person’s face, the different photographs of the famous person were presented on the screen at the same time. Participants selected the photograph(s) that they found most representative of the famous person, or they could indicate that they did not find any of the photographs representative.

As the total set of 468 enduringly famous persons was too long for one participant to work through, the total set was randomly split into two sets of 234 items. Subsequently, half of the participants saw one set of famous persons, and the other half of the participants saw the other set. The 234 famous names and the corresponding faces were presented in random order using Qualtrics EM software (32). Similar to step 1, data collection ended after each famous name/face had been evaluated by at least 20 older adults.

The photograph of each of the 468 famous persons was selected if participants judged them to be the most representative for that person. This selection was based on the number of times each photograph was judged as the most representative one. If different photographs of a famous person were chosen equally often, the photograph with an open-source CC license was chosen, as such photographs could be adapted, published, and shared. If none of the photographs had an open-source CC license, the photograph was selected that best matched the criteria in Table 3. Two famous persons were excluded after this step, as for them, most participants indicated that none of the photographs were representative. Consequently, a total of 1,720 photographs of famous faces remained, depicting 1,254 transiently and 466 enduringly famous persons.

## Step 3: Selecting famous faces that Dutch older adults can actually name

Steps 1 and 2 of this study resulted in a collection of 1,720 representative photographs of famous persons. The aim of step 3 was (e) to examine which famous faces Dutch older adults could name when presented with the photographs remaining after step 2.

### E. Selection of famous faces that older adults can actually name

In total, 452 older adults participated in step 3 (see Table 1 for sample descriptive statistics). Stimulus material in this step consisted of the photographs of 1,720 unique famous faces selected after steps 1 and 2. After a short practice session with five famous faces, participants completed the task. For each famous face, they saw the question: “Do you recognize this face?” (“Yes” or “No”). If they did not recognize the face (i.e., pressed “No”) or did not answer within 10 s, they automatically proceeded to the next famous face. If they recognized the famous face, they saw the next question: “Do you know this person’s name?” (responses could be: “Yes,” “It is on the tip of my tongue,” or “No, I do not know it”). If they did not answer within 15 s or answered anything other than “Yes,” they automatically proceeded to the next famous face. If they answered “Yes,” they were asked to enter the name of the person (without worrying about spelling).

As the total set of 1,720 famous faces was too much for one participant to work through in a single session, participants saw between 100 and 112 famous faces in one round. The 1,720 famous faces were divided into approximately similar sets with an equal number of faces per category. As some categories were bigger than others, this resulted in the different categories being unevenly represented in each set, e.g., one artist, 17 actors, 19 sportsmen, 9 public figures, 20 politicians, etc. Faces from each category were selected by pseudo random number generation in Excel (37). To be able to compare different sets that were completed by different participants, each set of 100–112 items had 24 overlapping items with only one other set. As an example, list 1 and list 2 had overlapping items and so did list 2 and list 3, but the overlap could not include the same items, such that list 1 and list 3 did not have overlapping items. After completing one set of famous faces, participants could choose to do another set. In that case, they would receive a new link via email. Participants could only see unique lists, that is, not do sets with overlapping famous faces. For each participant, the order of presenting the famous faces was randomized using Qualtrics EM software (32). Data collection ended when each of the 1,720 famous faces was evaluated by at least 35 older adults. The number of desired evaluations was higher for this step than for previous ones, because this step was the closest to a typical FFT and our eventual stimulus selection would be based on this.

Responses were defined as correct if the participant recalled the first and/or last name, or, if applicable, the correct artist’s name (e.g., Madonna). Person-identity semantic knowledge (PISK) about the famous person was not taken as a correct response for the reason that specifically face-name associations appear sensitive to the earliest stages of AD (38, 39). In line with this, participants were not instructed to fill out semantic information if they did not know the proper name. In 76.23% of correct responses, the respondent filled out both parts of the name. In the remaining 23.77%, only the first or last name was filled out. In 3.90% of correct responses, a correct artist name was filled out instead of a first and/or last name. Responses that did not exactly match the correct name were checked and scored manually by two independent student assistants, after which the scoring forms were combined by a member of the research team. Spelling mistakes, phonological, and/or alternative spelling (e.g., Dutch spelling of an international name or vice versa) did not matter and happened in 26.51% of responses, which were then classified as correct. An incorrect response included when a person responded to the famous face but did not fill out the name, gave a wrong or incomplete name, or (in)correct PISK (e.g., former president of the U.S.). A response was missing if the participant did not respond to the famous face although it was presented to them for at least 10 s.

For each of the 1,720 famous faces, the number of correct responses was calculated relative to the total number of responses. To illustrate, if a famous face had 40 responses and 12 were the correct name, the relative performance for that face was 30%. In addition, the item-rest correlation (i.e., corrected item-total correlation) was calculated as the correlation between the score on each item and the rest score (total score minus the item score). The item-rest correlation served as an item-quality index, as higher item-rest correlations within a test are known to result in higher coefficient α (40). As the item score is binary, a point biserial item-rest correlation is obtained (41).

**Figure 4:**
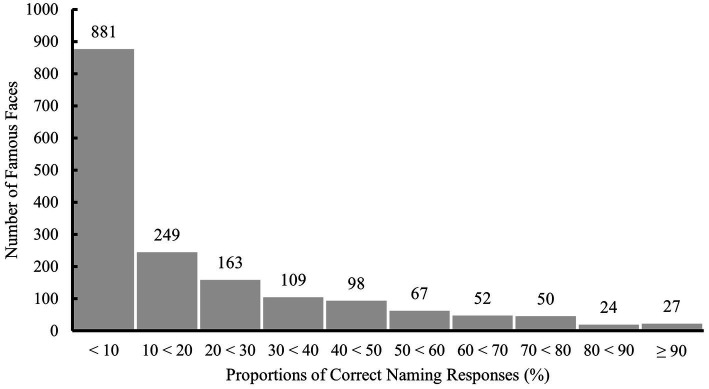
Distribution of proportions of correct naming responses to all 1,720 famous faces evaluated in step 3. Labels on top of each bar indicate the number of famous faces per interval.

**Figure 5:**
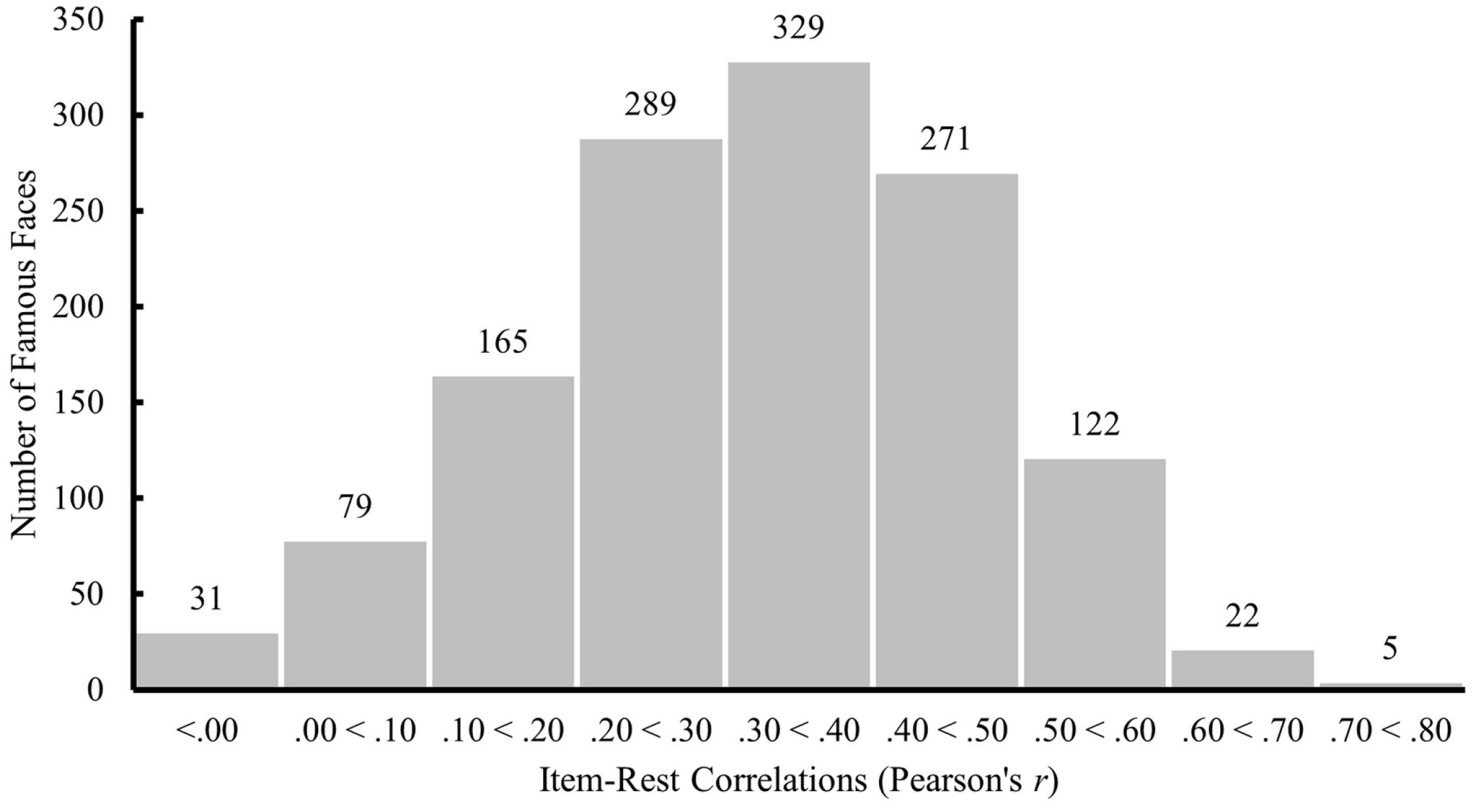
Distribution of item-rest correlations between participants’ scores on a famous face and total scores for all other famous faces in step 3. Labels on top of each bar indicate the number of famous faces per interval [Item-rest correlations could not be computed for 407 famous faces which were either correctly (one face) or incorrectly (406 faces) named by all participants].

Over all 1,720 famous faces, the mean percentage of participants who gave a correct response was 19.01% (*SD* = 23.51%, range: 0–100%, see Figure 4 for the distribution). The mean item-rest correlation was 0.32 (*SD* = 0.15, range: −0.19–0.78, see Figure 5 for the distribution). Relative performance of participants who had also completed steps 1 and/or 2 (*M* = 55.03%, *SD* = 21.99%) did not differ from that of individuals who had never seen the famous names before (*M* = 53.04%, *SD* = 20.72%), *t*(450) = −0.97, *p* = 0.33. There was no increase in performance for participants who did the task more than once, indicating that no learning effects occurred.

With the goal of only selecting famous faces that many participants could name while also avoiding ceiling effects, famous faces were selected for step 4 if at least 30% of the responses were correct. In addition, aiming for better item-quality, the point-biserial (item-rest) correlation had to be at least 0.20 (41). As a result, the 1,720 famous faces were reduced to 380.

## Step 4: Selection of open-source Dutch-famous faces test items

Steps 1 to 3 of this study resulted in 380 photographs of famous faces. In step 4, we aimed for a final selection of items with an open-source CC license. In order to be able to share both the task and stimulus material with other interested parties, an open-source CC license is required for each of the famous faces. The first aim of step 4 was (f) to replace famous faces with photographs with an open-source CC license if needed, that is, if they were not already open-source. This way, the stimulus material can be adapted, published, and shared in later stages. Subsequently, we aimed (g) to test all open-source famous faces in a sample of Dutch older adults, so that every participant saw each of the famous faces, of which some had been replaced by open-source photographs and were different from step 3.

### F. Selection of categories and decades and replacement with open-source photographs

Given the goal of creating multiple D-FFT versions for longitudinal assessment, only items in the four categories with the largest number of famous persons and six most represented decades were selected. These include Film & Theater, Politics, Singers & Musicians, and Sports, from the 1960s until the 2010s. In other decades and categories, there were not enough famous faces left to evenly distribute them over (approximately) 10 versions. This selection led to 284 remaining famous faces, of which 103 already had an open-source CC license, i.e., could be made both publicly available and also used in clinical practice during later steps of this project. At this point, the initial pool of famous persons was reduced from 4,574 famous names to 380 famous faces that older adults could actually name, and an attempt was made to find a new representative photograph of the 181 famous faces that did not yet have an open-source CC license. Replacement photographs needed to match the criteria in Table 3, taken in the same decade, and this time with an open-source CC license. This was successful for 117 items, resulting in 220 open-source famous faces. These 220 photographs were selected as the final stimulus material for the new D-FFT that is comprised of only open-source items. Note that ideally, only open-source items would have been selected from the beginning, but unfortunately, we did not come to this realization until step 4.

### G. Testing all open-source photographs in a sample of Dutch older adults

In total, 338 older adults participated in steps 4 and 5, of which 150 older adults were selected for step 4 by means of a random split (see Table 1 for sample descriptive statistics). As completing the D-FFT was part of a larger set of tasks, participants in steps 4 and 5 received 15 Euros as a reward for their participation. They could choose to receive the money on their personal bank account or donate it to a good cause; 52 percent of participants chose to donate.

After a practice session with 10 famous faces, participants completed the naming task. The D-FFT procedure was highly similar to step 3, except that the first question participants saw was: “Do you know this person’s name?” They could respond: “Yes,” “Yes, but it is on the tip of my tongue,” “No, but I recognize the face,” or “No, and I do not recognize the face.” If they did not answer within 15 s or answered anything other than “Yes,” they automatically proceeded to the next famous face. If they answered “Yes,” they were asked to enter the name of the person (without worrying about spelling). Participants were required to respond within 90 s after which the next famous face automatically appeared on the screen. In this way, participants were encouraged to give their first reaction to each face and not spend too much time deliberating on the answer. Subsequently, participants completed the recognition task, which was not used for stimulus selection due to near-ceiling effects and therefore is not described in further detail in the current paper.

**Figure 6:**
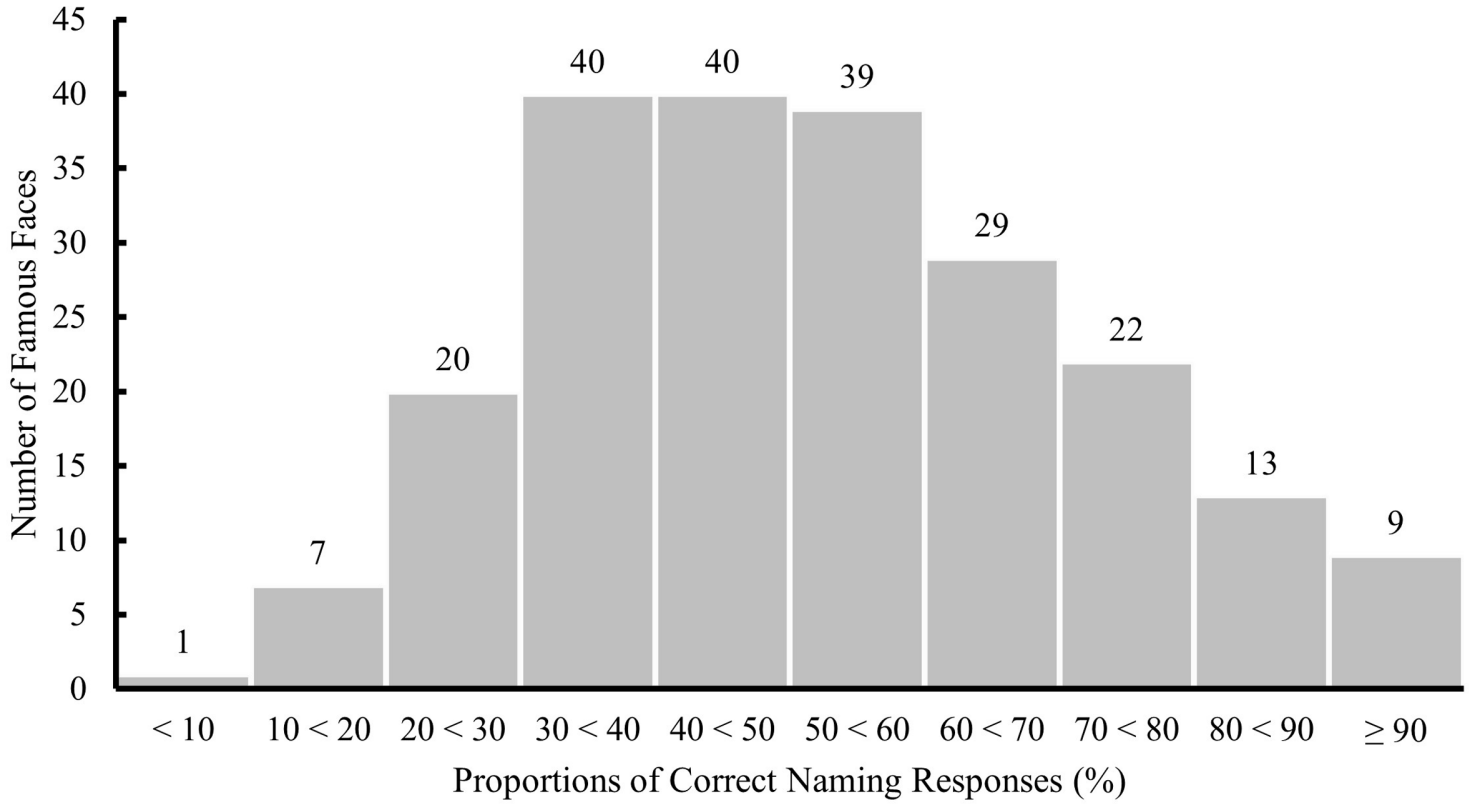
Distribution of proportions of correct naming responses to all 220 famous faces included in step 4. Labels on top of each bar indicate the number of famous faces per interval.

Participants completed all 220 D-FFT items, divided over two sessions that were completed within 1 week. The definition of a correct response and the calculation of performance were identical to step 3. Over all 220 famous faces, performance ranged from 8.05 to 98.66 percent (*M* = 52.25%, *SD* = 19.76%, see Figure 6 for the distribution).

## Step 5: Development of different Dutch famous faces test with equivalent mean difficulty levels

Step 4 of this study resulted in 220 open-source photographs of famous faces that had all been judged by 150 individuals (including 20 trial items). In step 5, we aimed to create short versions that were equivalent in terms of mean difficulty. Creation of short parallel versions is important in order to test individuals over time and also for use in clinical practice. The aims of step 5 were (h) to create parallel task versions of the D-FFT and (i) to test these versions in the remaining selection of 188 Dutch older adults in order to create tests with equivalent mean difficulty levels.

### H. Creation of different D-FFT versions each with an equivalent mean difficulty

The 220 famous faces were divided into 10 D-FFT versions, each comprised of 20 famous faces, and 20 items that can be used for practice. The D-FFT versions were developed based on the following three criteria, in order of importance: (1) mean performance in step 3, (2) distribution of the four categories, and (3) distribution of the six decades. The distribution of Dutch and international famous faces included as stimulus material in the 10 D-FFT versions is presented in Table 4.

**Table 4:** Distribution of Dutch and international famous faces included in the 10 D-FFT versions in step 5 (excl. trial items).

		Category								
		Dutch				International				
		F&T	M	P	S	F&T	M	P	S	Total
Decade	1960s	3	0	12	5	5	9	13	1	48
	1970s	6	3	7	3	1	7	3	2	32
	1980s	8	9	9	4	2	5	2	1	40
	1990s	1	0	2	1	1	5	0	0	10
	2000s	1	6	2	4	2	7	3	1	26
	2010s	9	8	6	4	1	11	1	4	44
Total		28	26	38	21	12	44	22	9	200

F&T, film & theater; M, music; P, politics; and S, sports.

The items were divided over 10 D-FFT versions following the procedure described in the Statistical Analysis section. 200 items were divided over 10 D-FFT versions of 20 items each, with 20 trial items. Mean performance over all participants on each version ranged between 51.16 and 53.71 percent (*M* = 52.42%, *SD* = 0.97%), based on the 150 participants in step 4. All versions had four items in the category Film & Theater, seven items in Politics, six items in Singers & Musicians, and three items in Sports. All versions had eight items from the 1960 and 1970s, four items from the 1980s, one item from the 1990s, and seven items from the 2000 and 2010s.

### I. Comparison of the D-FFT versions to ensure equivalent mean difficulty

The 10 D-FFT versions were tested in the remaining selection of 188 Dutch older adults (see Table 1 for sample descriptive statistics). As this selection resulted from splitting the total sample of 338 individuals who participated in steps 4 and 5, the D-FFT procedure was identical to step 4.

**Figure 7:**
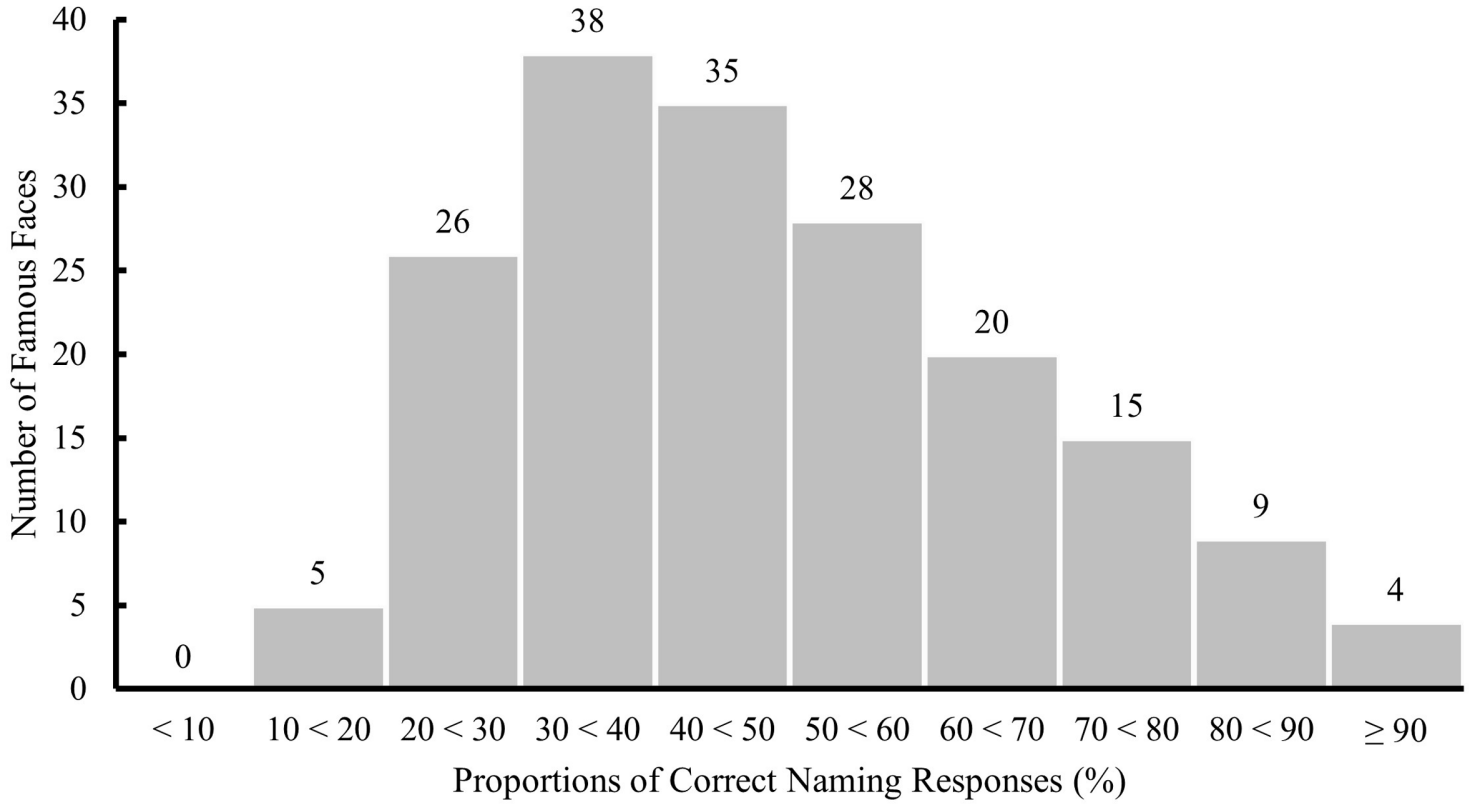
Distribution of proportions of correct naming responses to the 180 famous faces included in nine D-FFT versions in step 5 (excluding trial items and removed version). Labels on top of each bar indicate the number of famous faces per interval.

Equivalence of the 10 D-FFT versions was assessed by means of a repeated measure analysis as was described in the section Statistical Analysis. At the 0.05 level of significance, the omnibus F-test for testing the null hypothesis of equivalence of the proportion of correct naming responses was significant: *F*(9, 1,683) = 2.82, *p* = 0.003. Using Tukey’s procedure for all pairwise comparisons, only the difference between the extremes was significant. Therefore, we removed the version with the highest mean and reassessed equivalence. For the remaining nine versions, the null hypothesis that all means are equal against the alternative that at least one pair has different means, could not be rejected at the 0.05 level of significance: *F*(8, 1,496) = 1.89, *p* = 0.058. Over the 180 famous faces that were included in the nine D-FFT versions (i.e., excluding the 20 trial items and the removed version), performance ranged from 12.97 to 98.94 percent (*M* = 49.10%, *SD* = 19.49%, see Figure 7 for the distribution).

**Figure 8:**
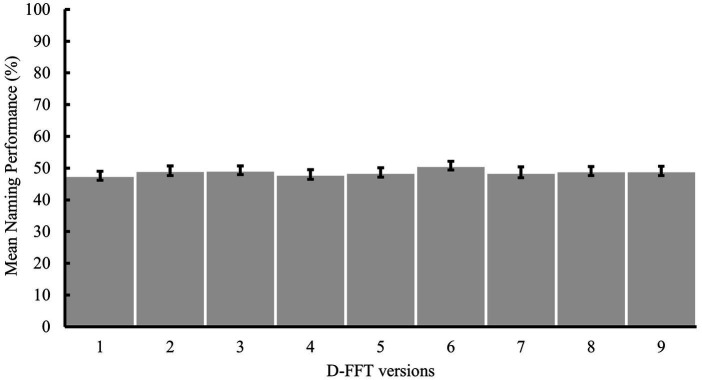
Mean naming performance on the nine D-FFT versions in step 5 with error bars representing the standard errors of the means.

Hence, nine of the D-FFT versions, all matched on the number of items per category and decade, were of equivalent mean difficulty. An overview of mean performance over the nine D-FFT versions is presented in Figure 8. The cumulative number of famous faces per proportion of correct naming responses to the 20 famous faces in each D-FFT version, including mean naming performance and standard deviations, is presented in the Supplementary material.

## General discussion

The aim of the current study was to explore the possibility of developing a Famous Faces Test specifically for older adults living in the Netherlands. Previous FFTs have generally not been developed for specific populations so they tend to be unstandardized and not generalizable. In pilot work conducted in our lab, a generic FFT was indeed found to be unsuitable for our population of interest, mainly due to the stimulus material used in previous studies. A new test was required. We also wanted to develop enough, short versions which we could use to (a) compare mean performance between the normal-aged controls and a clinical population and (b) create personalized norms for the normal aged controls that could be used to compare performance within the clinical population. The current article outlines the five steps taken to select famous faces in order to design and develop nine short versions of the Dutch FFT for older adults (60+) that are equivalent in terms of mean difficulty.

The current study has several unique strengths. To the best of the authors’ knowledge, this was the first time a Dutch FFT was developed specifically for older adults living in the Netherlands and also the first time an FFT was developed in a systematic, replicable manner. Starting with a collection of over 4,500 famous names in step 1, the current study produced nine short D-FFT versions of equivalent mean difficulty and balanced in terms of stimulus characteristics [i.e., the decade(s) in which the famous people were famous and the category they belong to] in step 5, that can be used for cross-sectional comparison as well as subsequent longitudinal assessment of cognitively normal and clinical groups. The selection of stimulus material was based on names that were known by at least a proportion (30%) of older adults, with photographs from the decade when the person was famous that were voted most representative by the current sample. Researchers interested in developing their own FFT-stimulus material could follow the steps outlined here for the specific country and/or population that they are interested in. They could also avoid some of the pitfalls possible in selecting material for such a task (including for example starting with open-source material if they want to make their task more widely available). The current study resulted in nine short D-FFT versions which we will use in the next part of this larger, ongoing project to explore both group differences (specifically do people with preclinical AD and/or MCI differ from normal, age-matched controls on this task) and individual differences (specifically which personal characteristics impact performance on this task).

A standard FFT was found in four previous studies to be sensitive to preclinical AD as indicated by biomarker assessment and/or clinical diagnoses several years after initial testing (25–28). Hence, detecting even the smallest impairment in FFT performance may be the key to timely AD diagnosis or at least indicate that comprehensive testing (i.e., standard clinical workup) should be considered. As every step in the development of the Dutch FFT is described in this article in detail, the task can easily be updated for new generations, and it could feasibly be developed for other countries as well. Importantly, older adults enjoyed filling out the D-FFT, as indicated by their frequent positive informal feedback and by step 3 where some participants enjoyed doing the task so much, they completed up to 11 rounds. Attrition rates are high for older adults in most longitudinal studies, with up to 29% older adults lost per wave, even when those lost to death and illness are excluded (42, 43). Attrition rates in future studies designed to track within-person trajectories over populations of older adults could be reduced when participation enjoyment is high. We will continue to assess this important aspect in future studies using the D-FFT. Lastly, because all famous faces have an open-source CC license, they can be published and shared with other researchers and health care professionals, even in their adapted, standardized formats.

Some limitations are however also worth mentioning. A major limitation of this study and approach is of course that the entire process including the selection of new photographs of famous faces will need to be repeated at least every decade in order to remain up to date with what is current in the world of politics, film and theater, sports, and music. Highly educated people were overrepresented in all five steps in the current study, i.e., between 68 and 80% had either finished high-level secondary education or had even obtained a university degree. As highly educated people perform better than those with less education in the naming of famous faces (44), difficulty levels of the Dutch FFT may currently be underestimated. Future data collection should ideally focus on a more representative sample, to optimize understanding of how individual differences, including educational level, affect performance on the D-FFT. Furthermore, although the nine versions were of equivalent mean difficulty, individual differences that potentially impacted performance have not yet been examined. Although a 30% cut-off criterion was used in steps 1 and 3, this was not applied in the last steps, which resulted in a recall performance below 30% for 31 items in step 5. However, performance was not below 10% for any of the famous faces and the 31 items are distributed over the nine D-FFT versions. At the individual level, these more difficult famous faces may be helpful when testing people who perform at a high level in order to avoid ceiling effects. Furthermore, because there are so few of these items, it is not expected that they will cause floor effects.’

In the next step of this ongoing project, the comparability of the nine D-FFT versions will be further explored considering the effects of personal characteristics, such as demographics (i.e., age, gender, and education) and other background information, including overall cognition-related (e.g., cognitive reserve) and D-FFT-specific variables (e.g., participants’ interests in the four different categories). Developing norms for our healthy older adults based on a range of such person-characteristics, as well as item-characteristics (e.g., the decades and categories in which celebrities occurred, their gender and nationality) will be useful to compare performance on the same FFT within clinical groups in a later phase of this project. In addition, we will examine in the next steps of this ongoing project whether a temporal gradient, as has been found in previous studies exploring preclinical AD (26, 29) is apparent or not in the D-FFT performance of CN older adults. As such, we aim to gain more insight into memory function in different stages of the AD continuum and specifically explore how to distinguish preclinical AD from normal aging. We will examine other response options, such as tip-of-the-tongue experiences (TOT), as well as the multiple-choice recognition task that was included in our D-FFT but not used for stimulus selection due to near-ceiling effects. We expect that this may be especially informative in individuals diagnosed with subjective cognitive complaints, MCI, and/or early AD (45). The D-FFT will also be compared to traditional neuropsychological tests within the same clinical population at a later stage in this ongoing project. Does our test perform as well as (or better) in classifying patients into the three clinical groups; namely preclinical AD or those who have subjective cognitive complaints (SCC), MCI and/or early AD? The nine versions of the D-FFT will also be used to explore group differences between normally aging and clinical populations. Standardizing the FFT, assessing background variables and testing these in both normally aging and clinical populations would be a major step forward in this field.

### Conclusion

This article describes the steps taken to select stimulus material for the first Dutch FFT, specifically for older adults. The systematic collection and selection of famous faces through five steps led to nine short D-FFT versions of equivalent mean difficulty. These nine D-FFT versions allow for the comparison of cognitively normal older adults and clinical populations by means of a short, enjoyable task that can be performed at a time and location convenient for the participant. Given that the standard FFT appears to be sensitive to preclinical AD (25–28), the development of a standardized FFT specifically for Dutch older adults may be the next step toward timely AD diagnosis.

## Data availability statement

The raw data supporting the conclusions of this article will be made available by the authors, without undue reservation.

## Ethics statement

The studies involving human participants were reviewed and approved by the ethical review board of the Tilburg School of Social and Behavioral Sciences at Tilburg University. The participants provided their written informed consent to participate in this study.

## Author contributions

EE, KD, RM, and YB contributed to the conception and design of the study. EE coordinated the data collection, organized the database, and wrote the first draft of the manuscript. EE and KD performed the statistical analysis. KD, RM, and YB critically revised the manuscript for important intellectual content. EE, RM and YB wrote the sections of the manuscript. All authors contributed to the article and approved the submitted version.

## Funding

This project is funded by a PhD grant awarded by the Herbert Simon Research Institute, Tilburg School of Social and Behavioral Sciences and Stichting Universiteitsfonds Tilburg.

## Conflict of interest

The authors declare that the research was conducted in the absence of any commercial or financial relationships that could be construed as a potential conflict of interest.

## Publisher’s note

All claims expressed in this article are solely those of the authors and do not necessarily represent those of their affiliated organizations, or those of the publisher, the editors and the reviewers. Any product that may be evaluated in this article, or claim that may be made by its manufacturer, is not guaranteed or endorsed by the publisher.
