# Supplementary material for: Stimulus material selection for the Dutch famous faces test for older adults
Source: Front Med (Lausanne). 2023 Apr 14;10:1124986. doi: 10.3389/fmed.2023.1124986 (PMC10140445; doi:10.3389/fmed.2023.1124986)
Supplement: Supplementary file 1 [file Table_1.docx]

Supplementary Material

Stimulus Material Selection for the Dutch Famous Faces Test for Older Adults

Evi H. T. van den Elzen^1,2^, Yvonne Brehmer^1,3^, Katrijn Van Deun^4^, Ruth E. Mark*^2^

*** Correspondence:** Ruth E. Mark: R.E.Mark@tilburguniversity.edu

# Supplementary Tables

**Supplementary Table 1**

*Overview of sources for famous names per category.*

| **Source** | **Year(s)** |
| --- | --- |
| *All categories*  Persons mentioned in Wikipedia’s Dutch decade overviews (e.g., https://nl.wikipedia.org/wiki/1950-1959) | 1940-2020 |
| Persons mentioned in Wikipedia’s Dutch yearly overviews (e.g., https://nl.wikipedia.org/wiki/1940) | 1940-2020 |
| *Artists*  Prix de Rome winners | 1940-2019 |
| *Film & Theatre*  Winners of the Oscars for best actor, best actress, and best film director | 1940-2020 |
| Winners of the Dutch ‘Gouden Kalf’ for best actor, best actor in TV drama, best actress, best actress in TV drama, and best film director | 1983-2020 |
| Winners of the Dutch ‘Gouden Televizier Ring Gala’ for best actor and best actress | 1964-2020 |
| Winners of the British Academy Film Award (BAFTA) for best actor in a main role, best actress in a main role, and best film director | 1952-2019 |
| Winners of the Golden Globe for best actor in a drama film (until 1950: best actor), best actress in a drama film (until 1950: best actress), best actor in a comical or musical film, best actress in a comical or musical film, and best film director | 1944-2020 |
| Comedians who did a Dutch New Year’s conference | 1954-2019 |
| Winners of the Dutch Poelifinario cabaret prize | 2003-2019 |
| Winners of the Dutch Neerlands Hoop cabaret prize | 2003-2019 |
| Winners of the Dutch National Schevening Cabaret prize | 1983-1998 |
| Winners of the Dutch Johan Kaart theatre prize | 1976-2019 |
| *Literature & Science*  Winners of the Dutch Gouden Penseel literature prize for best illustrated children’s book | 1973-2020 |
| Winners of the Dutch Gouden Griffel literature prize for youth literature | 1971-2020 |
| Winners of the Dutch Prijs der Nederlandse Letteren | 1956-2018 |
| Winners of the Dutch P.C. Hooft-prijs | 1947-2020 |
| Winners of the Dutch Libris Literatuur Prijs | 1994-2020 |
| Winners of the Dutch Boekenbon Literatuurprijs | 1987-2020 |
| *Public Figures*  Dutch NOS eight o’clock news presenters | 1982-2020 |
| Dutch NOS sports news presenters | 1984-2020 |
| Winners of the Dutch ‘Gouden Radio Ring’ for best radio DJ | 2006-2020 |
| Winners of the United Nations Prize in the Field of Human Rights | 1968-2018 |
| Winners of the Sacharov prize | 1988-2020 |
| TIME Persons of the Year | 1940-2020 |
| Winners of the Global Citizen Awards | 2010-2019 |
| Winners of the Nobel Peace Prize | 1944-2019 |
| Winners of the Dutch broadcaster of the year | 1991-2020 |
| *Singers and Musicians*  Winners of the Dutch Edisons | 1960-2020 |
| Winners of the Eurovision Song Festival | 1956-2019 |
| Dutch representatives at the Eurovision Song Festival | 1956-2019 |
| Winners of the Dutch Gouden Harp | 1962-2020 |
| Winners of the Grammy Award for best album of the year, best record of the year, best song of the year, and best new artist | 1959-2020 |
| *Sports*  Dutch medalists on the Olympic Games | 1948-2018 |
| Dutch Sportsmen of the year | 1951-2019 |
| Dutch Sportswomen of the year | 1959-2019 |
| Dutch talents of the year | 1973-2019 |
| Athletes mentioned in the Olympic Summer Game highlights (e.g., https://nl.wikipedia.org/wiki/Olympische_Zomerspelen_1948#Hoogtepunten) | 1948-2016 |
| Winners of the Ballon d’Or for best European soccer player (until 2006) and best soccer player in general (from 2007) | 1956-2019 |
| Winners of the FIFA world player of the year | 1991-2009 |
| Winners of the World Soccer Player of the Year | 1982-2019 |
| Winners of the Dutch ‘Gouden Schoen’ and premier league player of the year | 1963-2019 |
| Dutch Soccer talents of the year | 1984-2019 |
| Winners of the Tour de France | 1947-2020 |
| Winners of the Amstel Gold Race | 1966-2019 |
| Winners of Wimbledon | 1946-2019 |
| Winners of US Open | 1940-2020 |
| Winners of Australian Open | 1946-2020 |
| Winners of Roland Garros | 1941-2020 |
| Sports Illustrated Sportsmen of the Year | 1954-2017 |
| IAAF Athletes of the Year | 1988-2020 |
| KNAU Dutch Athletes of the Year | 1940-2019 |
| Winners of the Ard Schenk award for best Dutch male and female ice skater | 1990-2019 |
| Winners of the Egbert van ‘t Oever Encouragement Prize for Dutch ice skaters | 2002-2019 |

*Note.* Each described event was assessed for every occurrence between 1940 and 2020.

**Supplementary Table 2**

*Cumulative Number of Famous Faces per Proportion of Correct Naming Responses, Means, and SD for the 20 Famous Faces in Each of the Nine D-FFT Versions*

|  | **Proportion of Correct Naming Responses (%)** | | | | | | | | | | | |
| --- | --- | --- | --- | --- | --- | --- | --- | --- | --- | --- | --- | --- |
|  | **Cumulative Number of Items** | | | | | | | | | |  |  |
| **Version** | **< 10** | **< 20** | **< 30** | **< 40** | **< 50** | **< 60** | **< 70** | **< 80** | **< 90** | $\boldsymbol{\leq}$ **100** | ***M*** | ***SD*** |
| 1 | 0 | 0 | 4 | 9 | 12 | 15 | 17 | 19 | 20 | 20 | 47.6 | 20.0 |
| 2 | 0 | 0 | 1 | 6 | 11 | 15 | 20 | 20 | 20 | 20 | 49.2 | 21.1 |
| 3 | 0 | 1 | 6 | 8 | 11 | 13 | 14 | 18 | 20 | 20 | 49.3 | 18.8 |
| 4 | 0 | 1 | 2 | 9 | 13 | 16 | 18 | 18 | 20 | 20 | 48.0 | 21.0 |
| 5 | 0 | 1 | 4 | 10 | 12 | 14 | 15 | 18 | 19 | 20 | 48.6 | 19.9 |
| 6 | 0 | 1 | 2 | 6 | 11 | 15 | 17 | 18 | 19 | 20 | 50.8 | 18.7 |
| 7 | 0 | 0 | 3 | 7 | 10 | 16 | 18 | 20 | 20 | 20 | 48.6 | 23.2 |
| 8 | 0 | 1 | 4 | 7 | 13 | 15 | 16 | 17 | 19 | 20 | 49.1 | 19.5 |
| 9 | 0 | 0 | 5 | 7 | 11 | 14 | 17 | 19 | 19 | 20 | 49.1 | 20.5 |

*Note*. The mean (*M*) and standard deviation (*SD*) columns concern the percentage correct recall performance over the nine D-FFT versions.
